# Assessment of attitudes toward schizophrenia in tunisian family medicine residents

**DOI:** 10.1192/j.eurpsy.2021.1080

**Published:** 2021-08-13

**Authors:** B. Abassi, F. Fekih-Romdhane, H. Fayhaa, F. Ghrissi

**Affiliations:** 1 Psychiatry E, Razi hospital, Manouba, Tunisia; 2 Psychiatry E, Razi Hospital, La Manouba, Tunisia

**Keywords:** attitudes, schizophrénia, Tunisian family medicine residents

## Abstract

**Introduction:**

Assessing the attitudes of family medicine residents toward schizophrenia is of greatest concern since family physicians potentially have a key role in identifying the signs and symptoms of schizophrenia at earlier stages and in engaging young people in treatment, especially in low- and middle-income countries.

**Objectives:**

We aimed to investigate attitudes towards schizophrenia in a group of Tunisian family medicine residents, and to examine the link between these attitudes and help-seeking intentions in this group.

**Methods:**

This was a cross-sectional survey. A 18-item questionnaire concerning attitudes toward schizophrenia was used.

**Results:**

A total of 88% participants have reported favorable help-seeking intentions. In total, 48.4% of residents would oppose if one of their relatives would like to marry someone who has schizophrenia, and 37.1% of them would not like to have a neighbor with schizophrenia. Only about a half of residents agreed that “schizophrenia has the chance of recovery”, and 68.8% thought that “schizophrenia can be treated”. Pearson correlations found a significant negative relationship between age and social distance in residents (p<.001). Year of residency was significantly associated with attitudes toward schizophrenia, with more unfavorable attitudes in third-year residents (p=.042). After controlling for potentially confounding sociodemographic variables, help-seeking intentions did not contribute to the prediction of attitudes toward schizophrenia in the residents.

**Conclusions:**

Implementing anti-stigma programs in medical schools may help improve future physicians’ attitudes and prepare them to provide primary mental health care to young help-seekers with psychosis should be given priority attention.

